# Ulcerative colitis with mucosal lesions in duodenum: Two case reports

**DOI:** 10.1097/MD.0000000000015035

**Published:** 2019-04-05

**Authors:** Muran Li, Yandi Liu, Jifang Cui, Hai Qin, Yang Shi, Shiwu Zhang, Yongjie Zhao

**Affiliations:** a Department of Gastroenterology; b Department of Colorectal Surgery; c Department of Pathology; d Department of General Surgery, Tianjin Union Medical Center, Tianjin, China.

**Keywords:** duodenal, total colectomy, ulcerative colitis

## Abstract

**Rationale:**

Ulcerative colitis (UC) is a chronic, nonspecific, inflammatory disease of the colon. Colorectal is the main target organ of UC, while other digestive tract involvement is rare. This report describes 2 rare cases of duodenal mucosa lesions in patients with UC after total colectomy.

**Patient concerns:**

In case 1, a patient of 45-year-old with intermittent diarrhea and bloody mucosanguineous feces who was diagnosed as UC, revealed diffuse erosive ulcers in the descending duodenum through gastroscopy after total colectomy. In case 2, a 55-year-old Chinese female with UC, aggravated to colon cancer and received total colectomy. Eighteen months after surgery, the patient was admitted to hospital following upper abdominal pain and acid regurgitation. A gastroscopy found inflammation in the descending part of the duodenum.

**Diagnosis:**

UC, duodenal mucosa lesions

**Interventions:**

In case 1, the patient was treated with oral mesalazine (1 g/tid) and hydrocortisone (0.3 g/d) but symptoms did not improve, and the treatment was changed to oral methylprednisolone (0.6 g/d) and a hydrocortisone enema (0.1 g/late). Finally, the patient underwent a total colectomy and ileostomy. In case 2, the patient was treated with sulfasalazine, mesalazine, and intermittent hormone enemas. A total colectomy and ileostomy were performed with the patient after diagnosed as colon cancer. After surgery, the patient received N1-(2 tetrahydrofuryl)-5-fluorouracil (FT-207), 8 g, 300 mg, and 100 mg oxaliplatin chemotherapy, and biologic therapy.

**Outcomes:**

In case 1, the patient presented with duodenal necrosis and died of septic shock. In case 2, the patient recovered well without recurrence by taking proton pump inhibitor.

**Lessons:**

The occurrence of UC related ulcerative gastroduodenal mucosal lesions may be associated with progressing UC or total colitis that does not respond to hormone therapy, leading to requirement of total colectomy.

## 1 Introduction

Ulcerative colitis (UC) is a chronic, nonspecific, inflammatory disease of the colon. The etiology is unknown, and an increasing number of studies have confirmed that the disease is related to genetic, autoimmune, and infection factors.^[1]^ UC often presents with abdominal pain, diarrhea, and hematochezia. It is important to exclude infection as the etiology of UC. Colorectal is the main target organ of UC, while other digestive tract involvement is rare. In recent years, it has been reported that patients with UC may also develop complications with gastric and duodenal mucosal lesions. These complications may be associated with colorectal resection in patients with UC, but the pathogenesis is not yet clear.^[2,3]^ Our report describes 2 cases of patients with UC with duodenal descending lesions after colorectal resection and demonstrates that the clinical and pathologic features of duodenal mucosal lesions are very similar to those of UC.

This case report was approved by the ethics committee of the Tianjin Union Medical Center, Tianjin, China, and the informed consent form was signed by patient.

## 2 Case report

### 2.1 Case 1

A 45-year-old Chinese male, suffering from intermittent diarrhea and bloody mucosanguineous feces for 3 years and aggravation for 4 months was referred to our department after 1 week of recurring low-grade fever. The patient had been experiencing diarrhea with dull pain in the left abdomen and a loose, visible mucus, and purulent sanguineous stool, 3 to 4 times a day for 3 years. He had initially responded to combined therapy with oral mesalazine (1 g/tid), local mesalazine suppository (0.5 g/tid), followed by oral triplex live bacteria capsules (630 mg/tid). The patient responded positively to this treatment for 3 months, passing yellow stool 1 to 2 times daily, with no mucus, pus, or abdominal pain. However, for 4 months before admission, the patient had diarrhea 6 to 7 times a day, with bloody mucosanguineous feces, and abdomen pain, with no response to local mesalazine suppository (1 g/tid). He had a body temperature of 37.4°C for 1 week before admission, had smoked for 20 years, and had a history of hepatitis B (HBV). A physical examination found no obvious abnormality of the abdomen.

**Figure 1:**
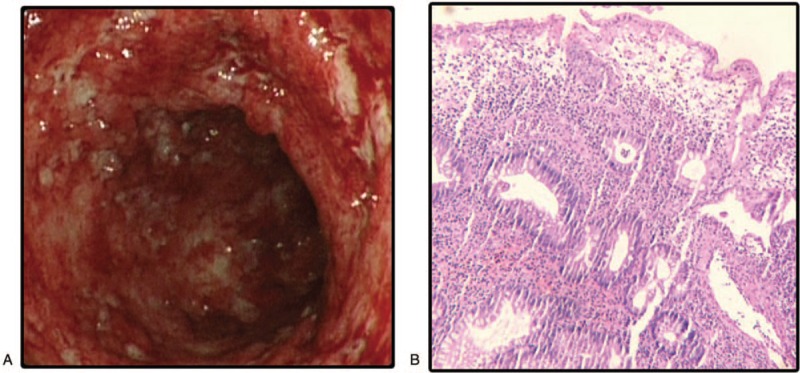
Colonoscopy and histologic findings in colon. (A) The diffuse erosive ulcers in descending colon, sigmoid colon, and rectum. (B) Inflammation in the mucous membrane of colon and rectum; a large number of lymphocytes, plasma cells, neutrophils, and a few eosinophils infiltrated the lamina propria; an occasional cryptic abscess, mild atypical hyperplasia of some glands.

All abdominal enhanced CT showed lesions of distal colon, descending colon, sigmoid colon, and rectum consistent with UC, as well as reactive lymph node hyperplasia. The patient was diagnosed with UC based on a colonoscopy (Fig. 1A) and histology of a biopsy (Fig. 1B). The colonoscopy showed diffuse erosive ulcers in the descending colon, sigmoid colon, and rectum. A pathologic biopsy showed acute and chronic inflammation of the mucous membrane with superficial erosion, the occasional cryptic abscess, mild atypical hyperplasia of some glands, and infiltration of lamina propria lymphocytes, plasma cells, neutrophils, and a few eosinophils.

The patient received oral mesalazine (1 g/tid) and hydrocortisone (0.3 g/d) but symptoms did not improve. After 10 days, the treatment was changed to oral methylprednisolone (0.6 g/d) and a hydrocortisone enema (0.1 g/late). The patient underwent a total colectomy and ileostomy because he did not respond to treatment after 2 weeks and had HBV replication. Furthermore, 30 mg Urbason was given for symptom treatment postoperation.

**Figure 2:**
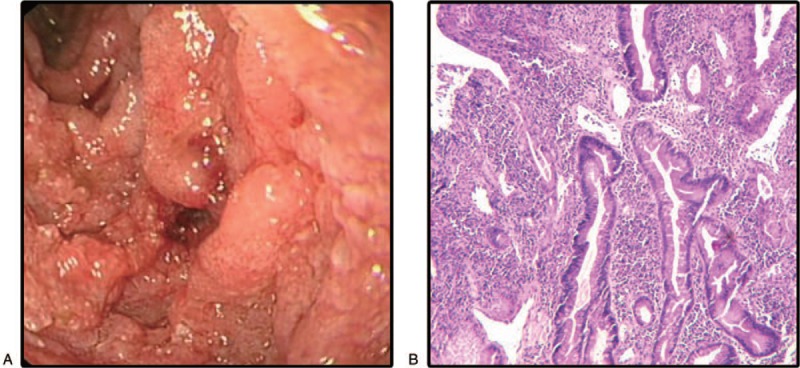
Colonoscopy and histologic findings in duodenum. (A) The diffuse erosive ulcers and bleeding in the descending duodenum. (B) Inflammation in the mucous membrane with superficial erosion.

Gastroscopy was performed 12 days after the operation, revealing no lesion in the gastroduodenal area, because approximately 600 mL of tartar-like stool was found from anastomosis. Twenty days later, a 2nd gastroscopy was performed, revealing diffuse erosive ulcers and bleeding in the descending duodenum. A pathologic biopsy showed acute and chronic inflammation of the mucous membrane with superficial erosion (Fig. 2). An arteriography of the gastroduodenal artery was performed and can be seen the contrast agent spillover and intestinal canal staining in the branch of the gastroduodenal artery, and be considered the arterial branch of the pancreas and duodenum. Vital signs gradually steadied after embolization. After 23 days, the patient presented with hematemesis, abdominal pain, and low fever. Abdominal drainage was yellow-green, and abdominal CT examination indicated intestinal perforation. The anterior wall of the duodenal descending segment was necrotic and defect about 2 × 3 cm during surgery. Following surgery, the patient died of septic shock.

### 2.2 Case 2

The patient, a 55-year-old Chinese female, presented with intermittent diarrhea with little bloody mucosanguineous feces, 4 to 6 times a day, and pain in the lower left abdomen. The symptom could relieve for 10 years. An endoscopy showed rough and eroded mucosa of the rectum and sigmoid colon, the vascular network was not clear, there were a few pus secretions, and 3 polypoid protrusions were seen in the sigmoid colon. An endoscopic polypectomy was performed in 2010, and postoperative pathology revealed inflammatory polyps. The patient was diagnosed with UC according to histopathologic criteria. She received intermittent oral sulfasalazine and mesalazine for maintenance treatment, intermittent hormone enemas, and Chinese medicine. This treatment relieved symptoms, but episodes of diarrhea continued.

**Figure 3:**
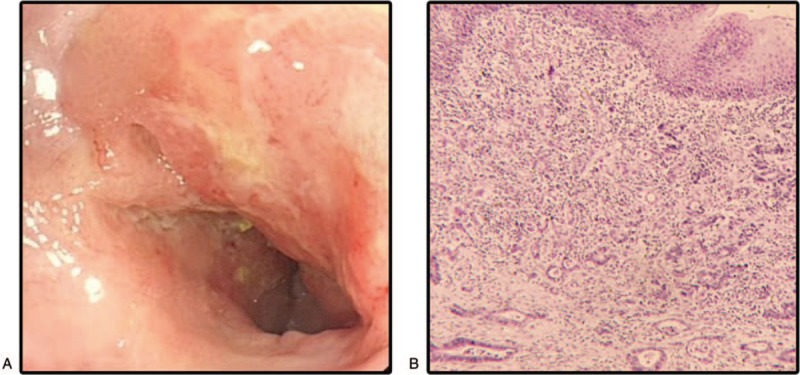
Colonoscopy and histologic findings in colon. (A) Stenosis of 4 cm at the entrance to the large intestine, mucosa was rough and bleeding easily, and the endoscope could not pass through the colon. (B) Rectal adenocarcinoma.

The patient experienced aggravated symptoms from 2013. Endoscopic examination revealed stenosis of 4 cm at the entrance to the large intestine, mucosa was rough and bleeding easily, and the endoscope could not pass through the colon. Furthermore, the pathologic biopsy revealed rectal adenocarcinoma (Fig. 3). On January 5, 2013, a total colectomy and ileostomy were performed with the patient under general anesthesia. The postoperative histology biopsy showed: the ileum 4 cm in length and 4 cm in diameter; the total colorectal anal canal 84 cm in length and 3.5 to 7 cm in diameter; chronic inflammation present in the mucous tissue along the 35 cm ranging from the pectinate line; dilated and congested interstitial blood vessels; erosion ulcers and recess abscesses; focal glands showing atypical hyperplasia; and increased submucous fibrous tissue. The medium and low differentiated adenocarcinomas were found 3 cm from the pectinate line. The volume of the adenocarcinoma was 6 × 3.5 × 0.5 to 0.8 cm, and there was invasion of the fibrous membrane, the recidivist nerve, and the tumor thrombus in the vascular canal. There was no cancer invasion on both sides and metastatic carcinoma in the mesenteric lymph nodes. The clinical stage was T3N2Mx, Dukes C stage. Combined with the clinical history, these factors indicate carcinogenesis in UC. After surgery, the patient received N1-(2 tetrahydrofuryl)-5-fluorouracil (FT-207) and 8 g, 300 mg, and 100 mg in oxaliplatin chemotherapy, and biologic therapy.

**Figure 4:**
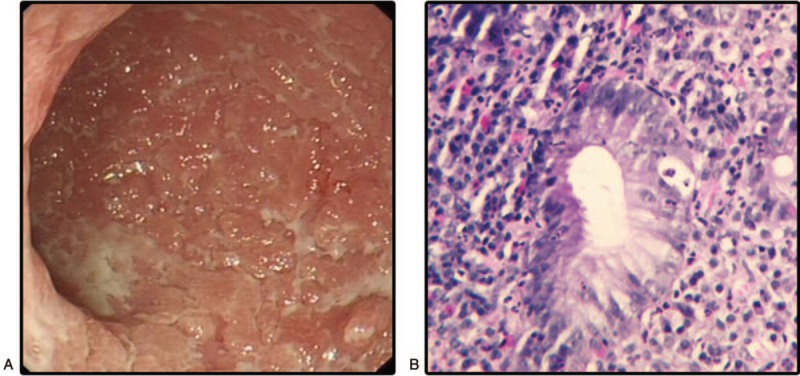
Colonoscopy and histologic findings in duodenum. (A) Diffuse thickening of the muscularis of duodenal, post and descending mucosa, mucous membrane rough and erosive, normal submucosa and inherent muscularis structure, narrowing of the descending intestinal cavity and nonspecific inflammatory changes. (B) Mucosal superficial ulcers, a large number of acute and chronic inflammatory cell infiltration, and moderate hyperplasia of glandular.

Eighteen months after surgery, the patient was admitted to hospital following upper abdominal pain and acid regurgitation. A gastroscopy found inflammation in the descending part of the duodenum. Endoscopic ultrasonography revealed: diffuse thickening of the muscularis mucosa in the post and descending duodenum; rough and eroded mucous membrane, normal submucosa and inherent muscularis mucosa structure, narrowing of the descending intestinal cavity, and nonspecific inflammatory changes. The endoscope found that duodenal sphere and the descending stenosis, making it difficult to insert the mirror; mucous membrane edema; extensive ulcers, white mass scattered in the polypoid protrusion, and a lesion approximately 6 cm in length over the narrow segment and the smooth mucous membrane of the distal intestinal mucosa. Pathologic biopsy revealed mucosal superficial ulcers, a large amount of acute and chronic inflammatory cell infiltration, and moderate hyperplasia of the glandular epithelium (Fig. 4). The patient recovered well without recurrence by taking proton pump inhibitor.

## 3 Discussion

The main predilection site of UC is colorectal cancer, while UC complicated with upper gastrointestinal tract gastroduodenal mucosal lesions is rare. An earlier study reported that 2 patients with UC developed duodenitis after total colectomy.^[4]^ Subsequently, other studies have reported the same complication,^[5–9]^ and different scholars have defined the complication in recent years. Japanese scholars defined it as UC-related gastroduodenitis, or UC-related ulcerative gastroduodenal mucosal lesions (UGDL),^[2]^ and the diagnostic criteria are: lesions that do not respond to standard treatment for ulcers, such as H2 blockers and proton pump inhibitors, and lesions that are sensitive to medication used to treat UC, such as steroids and mesalazine. The histopathologic manifestations in all studies were similar to those of UC, including diffuse inflammatory cell infiltration, crypts, crypts, and crypts. Endoscopy in 2 studies showed a rough, eroded, granular mucous membrane, and multiple ulcers.^[10]^ The study reported that 2 patients underwent total colectomy after failure to respond to standard treatment. Postoperative endoscopy of the duodenum found erosion of the mucosa and extensive ulcers. A biopsy revealed that a large number of inflammatory cells had infiltrated the duodenum, along with shallow ulcers. The progress of the disease is consistent with the diagnostic criteria of UGDL according to the results of endoscopic and pathologic biopsy. Patient in case 1 performed intestinal perforation followed surgery and died of septic shock. Patient in case 2 recovered well without recurrence by taking proton pump inhibitor.

There is no definitive conclusion about the exact pathogenesis of UGDL. According to previous reports, 15 patients with UGDL had colitis, and 14 of them needed total colectomy.^[10]^ In this report, the 2 patients with UC underwent a total colectomy after ineffective hormone therapy, then carcinogenesis in UC. The UGDL phenomenon occurred after the operation; therefore, it may be associated with total colectomy. It is concluded that if the active stage of severe UC is progressing to total colitis, then the patients with UC who needs a total colectomy has a higher probability of complications with UGDL. As for exploring the relationship between the occurrence of UGDL and total colectomy, some scholars believe that the treatment of a lower dose of prednisolone after surgery may increase the risk of inducing UGDL.^[10]^ Recent studies on the pathogenesis of inflammatory bowel disease suggest that chronic inflammation may be caused by the body's cellular immune response to certain bacteria.^[11]^ In the pathologic analysis of patients with UC, it was found that there were similar immune molecules in the upper gastrointestinal tissue and the colon tissue, and the CCR3+ and CD62L-activated Th2 cells in the upper digestive tract, may be derived from the tissue of the colonic mucosal lesion.^[12]^ Based on this theory, UGDL may arise from genetic susceptibility to a disorder of the body's immune response, resulting in an excessive autoimmune reaction to bacterial antigen, leading to the recruitment of memory T cells in the colorectal mucosa to the upper digestive tract.^[13]^ In addition, current studies suggest that *Helicobacter pylori* is not necessarily related to the occurrence of UGDL.^[2,14]^

Because UGDL and UC have similar histologic features, previous reports have shown that many patients with UGDL, especially those with mild symptoms, respond positively to the treatment of UC with drugs such as sulfasalazine or mesalazine.^[15,16]^ For some patients with severe UGDL, either sulfasalazine or steroid hormones could not control the condition effectively, and the tumor necrosis factor (TNF) antagonist Infliximab (IFX) or a calcium antagonist could be more effective in preventing the condition from deteriorating.^[17,18]^ To date, it is considered that early diagnosis with application of sulfasalazine or mesalazine is a quick and effective way to improve UGDL symptoms, and steroids could be used as a 2nd-line therapy for UGDL and the TNF antagonist IFX or a calcium antagonist for patients with rapid progress and hormone insensitivity.

With the increasing number of upper gastrointestinal mucosal lesions in patients with UC, we should pay attention to the pathogenesis and treatment of UGDL. The present study suggests that the occurrence of UGDL may be associated with progressing UC or total colitis that does not respond to hormone therapy, leading to requirement of total colectomy. The treatment of UGDL is similar to that of UC, with administration of sulfasalazine, mesalazine, or steroid hormones. Patients with severe UGDL should be also treated with TNF antagonist IFX or calcium antagonists.

## Author contributions

**Investigation:** Yandi Liu, Jifang Cui.

**Resources:** Hai Qin, Yang Shi, Shiwu Zhang.

**Writing – Original Draft:** Muran Li.

**Writing – Review & Editing:** Muran Li, Yongjie Zhao.
